# Central nervous system vascular malformations: A clinical review

**DOI:** 10.1002/acn3.51277

**Published:** 2021-01-12

**Authors:** Behnam Sabayan, Christina Lineback, Anand Viswanathan, Thabele M. Leslie‐Mazwi, Ali Shaibani

**Affiliations:** ^1^ Department of Neurology Northwestern University Feinberg School of Medicine Chicago Illinois USA; ^2^ Department of Neurology Massachusetts General Hospital Harvard Medical School Boston Massachusetts USA; ^3^ Departments of Neurosurgery and Neurology Massachusetts General Hospital Harvard Medical School Boston Massachusetts USA; ^4^ Department of Radiology Northwestern University Feinberg School of Medicine Chicago Illinois USA

**Keywords:** central nervous system, intracranial, prognosis, spinal cord, vascular malformation

## Abstract

CNS vascular malformation is an umbrella term that encompasses a wide variety of pathologies, with a wide range of therapeutic and diagnostic importance. This range spans lesions with a risk of devastating neurological compromise to lesions with a slow, static or benign course. Advances in neurovascular imaging along with increased utilization of these advances, have resulted in more frequent identification of these lesions. In this article, we provide an overview on definitions and classifications of CNS vascular malformations and outline the etiologic, diagnostic, prognostic, and therapeutic features for each entity. This review covers intracranial and spinal cord vascular malformations and discusses syndromes associated with CNS vascular malformations.

## Background

Vascular lesions of the brain and spinal cord are commonly encountered in clinical practice and can lead to diagnostic, prognostic and therapeutic challenges.^1^ Central nervous system (CNS) vascular malformations encompass a wide range of arterial and venous anomalies with various presentations, clinical course, and complication rates.^2^ Due to increased utilization of imaging techniques of the cranio‐spinal axis over the past decades, more vascular malformations are being detected. This necessitates an increased level of expertise with the diagnosis, characterization, and timely management of these lesions.^3^ The term malformation can imply a congenital (developmental) or an acquired lesion and both terms (malformation and lesions) have been used interchangeably.

The most common brain vascular lesions in adults are arteriovenous shunts and cavernous malformations with estimated detection rates of 1.0 and 0.5 per 100 000 adults per year, respectively.^4^, ^5^ Spinal cord vascular lesions are rare and spinal dural arteriovenous shunts account for more than 70% of these lesions.^6^ Patients with CNS vascular lesions can present with a variety of presentations from headache and seizure to isolated cranial nerve deficits and progressive motor and sensory alterations. This highlights the importance of high clinical suspicion and early detection to reduce future risk of complications. Clinicians frequently face questions about the use of antiplatelet and anticoagulation medications as well as the need for prophylactic measures such as administration of antiepileptic medications in this patient population. In this review, we will outline epidemiological, clinical, radiological, and therapeutic features of intracranial and spinal cord vascular malformations and discuss syndromes associated with CNS vascular malformations.

## Pathobioiology and categories of malformations

The key pathology underlying vascular malformations is impairment in integrity of capillary, venous, and arterial beds. This loss of integrity can be due to external causes such as mechanical injuries and/or defects in vascular development during angiogenesis, vessel growth, and maturation.^7^ Various factors such as vascular endothelial growth factors, fibroblast growth factors, platelet‐derived growth factors, and angiopoietins act in concert to regulate angiogenesis. Alterations in the complex interactions between these factors can lead to the development, progression, and regression of vascular malformations.^8^ In addition to a faulty angiogenesis, increasing evidence indicates that inflammation plays a major role in vascular dysmorphogenesis and changes in structural and functional properties of the vessels in the central nervous system.^9^

In the last decades, the genetic basis of several CNS vascular malformations has been further explained. Certain vascular malformations classically present in an autosomal dominant fashion such as familial cases of cavernous malformation, hereditary hemorrhagic telangiectasia, and capillary malformation–arteriovenous malformation as detailed later in this article. However, the vast majority of vascular malformation occurs sporadically. Several genetic variants have been identified that can render individuals susceptible to vascular malformation formation and complications.^10^ These genetic variants mainly regulate blood–brain barrier integrity, transforming growth factor‐β (TGF‐β) signaling pathway, local inflammation response, angiogenesis, and tissue remodeling. Endoglin, activin‐like kinase (ALK) receptor 1, somatic‐activating KRAS, and RASA‐1 gene are among the identified genes contributing to CNS vascular malformations.^8^, ^11^, ^12^

CNS vascular malformations can be classified based on various features including anatomical location, clinical presentation, radiological characteristics, histopathological findings, or hemodynamic status. In this review, we focus on the anatomic location (intracranial, spinal cord, and syndromic) as well as the clinical manifestations of each malformation and discuss risks of complications. Table 1 classifies the malformations based on their anatomic location and hemodynamic status.

**Table 1:** Categories of CNS vascular malformation.

	Hemodynamic	Common location(s)	Clinical presentations
Arterial/mixed with arterial components			
Arteriovenous malformations	High flow	Throughout CNS	Hemorrhage, seizure and headache
Dural arteriovenous shunts	High flow	Transverse or sigmoid sinus	Tinnitus Hemorrhage
Carotid cavernous fistula	High flow	Carotid cavernous fistula	Exophthalmos, cephalic bruit and conjunctival congestion
Vein of Galen Malformation	High flow	Vein of markowski	Newborn heart failure
Venous			
Cerebral Cavernous malformation	Low flow	Supratentorial	Seizure, incidental finding
Developmental Venous Anomalies	Low flow	Various locations	Headache, seizures
Sinus peri‐crani	Low flow	Frontal‐midline location	Cosmetic issues, headaches, tinnitus
Capillary			
Capillary telangiectasia	Low flow	Pons	Cranial nerve palsies

## Intracranial vascular lesions

### Arteriovenous shunts

#### Arteriovenous malformations

Arteriovenous (AV) malformations are classically considered to be congenital and comprised of feeding arteries, nidus, and draining veins.^13^ A key hallmark of this malformation is the lack of a capillary bed. These lesions are vascular shunts typically supplied by arteries that perfuse brain parenchyma (branches of the internal carotid or vertebrobasilar systems), though large or superifical lesions can occasionally recruit external carotid branches (e.g., middle or posterior meningeal artery). The tangle of vessels between the feeding arter(ies) and draining vein(s) is called the nidus. The nidus is characterized (by angiography) in two ways, diffuse or more commonly compact or “glomerular.” There is no brain tissue within the nidus of the glomerular AVM.^14^ Multiple hypotheses have been proposed on the etiology of AV malformations including; retained embryonic vascular connections, vascular changes due to inflammatory responses, and a poorly defined role for elevated vascular endothelial growth factor.^15^ There are contemporary reports of de novo formation postnatally and into adulthood with underlying “dysregulated angiogenesis” with genetic underpinning as the etiology.^16^, ^17^ There are various morphological subtypes including wedge‐shaped AV malformations – based on the cortex, near border zones which is a classic type, cylindrical or globoid type – restricted to the white matter which does not involve the surface of the brain and less common variants involving individual arteries rather than brain tissue.^17^, ^18^

**Figure 1:**
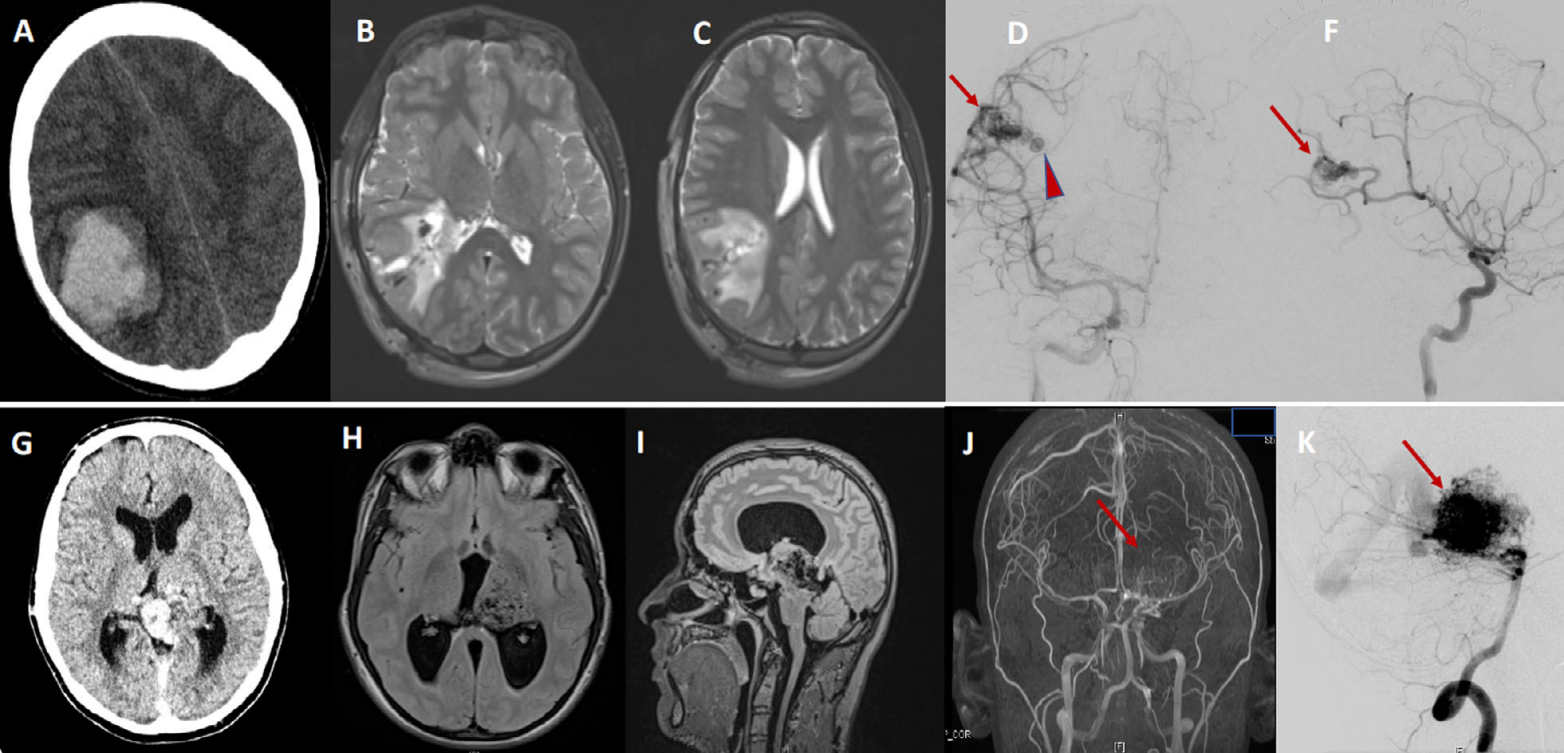
Top Row: A 12‐year‐old boy presenting with acute onset loss of consciousness, left hemiparesis, and left hemibody sensory loss. Axial noncontrast CT (A) showed acute right parietal intracranial hemorrhage with midline shift. Axial MRI T2W images postemergent evacuation of the hematoma, with resolution of mass effect (B, C). Right internal carotid artery angiogram demonstrating a small right parietal arteriovenous malformation (AVM) (arrow), with an intra‐nidal aneurysm (arrowhead) compatible with the rupture site (D, F). Bottom Row: An 18‐year‐old man with headaches, difficulty walking, and hearing a whooshing sound after taking a flight for the first time. Noncontrast CT (G) and brain MRI (H, I) showed a large left thalamic mass causing moderate obstructive hydrocephalus. MRA (J) showed a large vascular lesion with numerous enhancing tortuous vessels (arrow) in the left thalamus extending inferiorly to midbrain. Vertebral angiography (K) confirmed AVM with the dominant supply from the left posterior cerebral artery and deep venous drainage.

These high flow malformations are increasingly incidentally discovered (Figure 1). AV malformations are equally seen among the sexes and the most commonly affected age group is 20‐40 years. Older postmortem data suggested that approximately 10% of AV malformations become symptomatic during life. The most common presenting feature is hemorrhage, seen in up to 50% of cases^1^, ^19^, ^20^, ^21^ followed by seizures, headache, and focal neurologic deficits. Of hemorrhages – intraparenchymal hemorrhage is the most common complication followed by subarachnoid or intraventricular bleeding. Intracranial hemorrhage related to an underlying AVM in total represents 2% of all hemorrhagic strokes and is the leading cause of nontraumatic intracerebral hemorrhage in patients younger than 35 years.^4^, ^22^ It remains uncommon that patients present with multiple AVMs, but when it is the case, an underlying condition such as hereditary hemorrhagic telangiectasia should be considered.^23^

The current literature reports a 2‐4% annual risk of rupture.^13^, ^14^, ^20^ Variables that impact this risk include age, location, nature of the feeding arteries, and draining veins. The strongest predictor for hemorrhage is a history of bleeding in the past, with the estimated 6‐25% annual re‐bleeding risk within the first five years, with the first year carrying the highest risk.^20^, ^22^ Other lesion characteristics that may predict increase risk of bleeding include; single draining vein or deep venous drainage due to increased outflow impedance, location in posterior fossa, ventricular, periventricular, and deep cerebral regions. In addition, nidal aneurysms, venous stenosis or varices, and elevated pressure within the nidus or feeding arteries can increase the hemorrhagic complications.^22^ If none of these high‐risk characteristics are present, the risk of hemorrhage is estimated at less than 1%.^13^, ^22^

Cerebral proliferative angiopathy (CPA) was previously described as “diffuse AVM” and is a less common presentation than glomerular AVM. CPA is defined as a diffuse network of abnormal vessels surrounded by *normal* brain parenchyma. One hypothesis for the etiology of these lesions is chronic hypoperfusion.^24^ In a review of 1400 brain AVMs, only 3.4% were identified as CPA.^25^ Compared to more common glomerular AVMs, CPAs less frequently present with hemorrhage^25^

Pregnancy and puerperium may increase the risk of hemorrhage, however, this is an area of conflicting data with increased risk quoted from 0% to 8%.^26^ The impact of anticoagulation is also uncertain. There are no large randomized control trials to support higher rates of hemorrhage of AVMs for patients on anticoagulation or antiplatelet therapy. Generally, these lesions should not stop clinicians from treating patients who have medical indications for anticoagulation. However, it is noted that if rupture was to occur, being on anticoagulation may worsen outcomes.^27^ In a small retrospective study on 77 individuals, antiplatelet therapy was not associated with increased rupture rate.^28^

Imaging techniques for detailed characterization of AV malformations include MR angiogram, CT angiogram, and the gold standard imaging technique of digital subtraction angiography (DSA). Conventional angiography allows for better visualization of early venous drainage, the defining feature of AV malformations, which can assist in risk assessment. Additional neuroimaging modalities such as functional MRI allow for assessing structural brain alterations in relation to the malformation, with treatment implications.^29^

Understanding the pathophysiology, natural history, and characteristics predictive of the risk of rupture are all essential for clinicians when making appropriate therapeutic decisions. There are two main branches of therapy – intervention or medical management. Medical management, or observation, includes treating risk factors for rupture such as hypertension and smoking cessation, and treating symptoms of the lesion itself, including headache and seizures. Surgical and radiologic interventions include endovascular therapy, surgery, and radiotherapy alone or in combination.^1^ There are limited data comparing the medical versus interventional management options. The ARUBA clinical trial was designed to compare intervention versus medical management in patients with brain AVM.^30^ This was an un‐blinded, randomized trial of 223 patients. The trial ended early with the measured outcome of death or symptomatic stroke (intervention 30.7%, vs. medical 10.1%). There were several limitations to this study and caution should be exercised in the interpretation of the findings.^30^ The majority of patients in the intervention group underwent endovascular treatment (not considered to be the primary treatment mode in most major centers because of limited ability to achieve complete cure) and for about 20% of patient’s outcomes were not clearly reported. In addition, patients were not controlled for symptoms prior to intervention. In this study, participants were older white adults limiting the generalizability of the results. In 2016, a 5‐year follow‐up data showed favorable outcomes in the medical group again, with exception of radiosurgery which showed a benefit of treatment.^31^ Recently the investigators extended the follow‐up time and again data show that interventional management might pose extra risk in the long‐run.^32^

#### Dural Arteriovenous shunts

**Figure 2:**
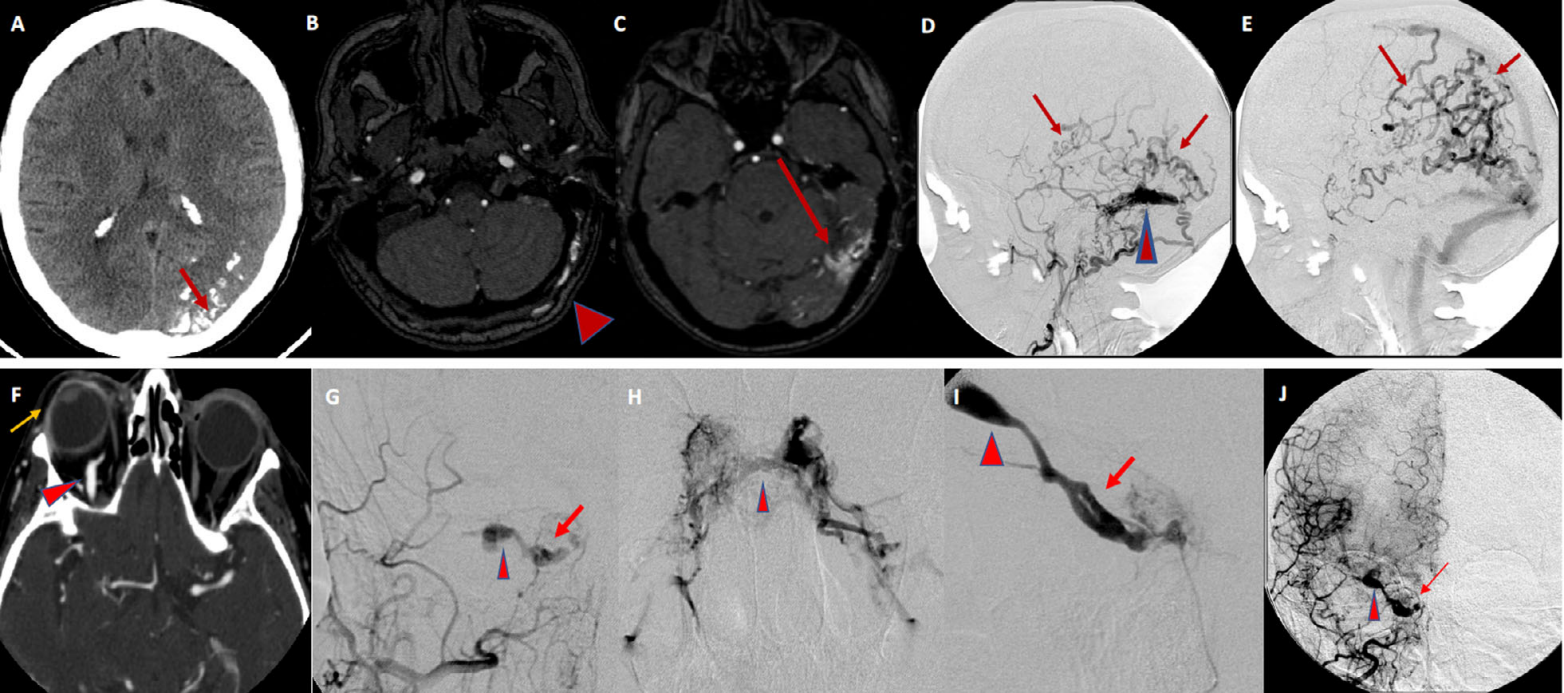
A middle‐aged man with seizure, headache, and right visual field disturbance. Axial noncontrast head CT demonstrating calcification in the walls of the left occipital veins (arrow) indicating long‐standing venous hypertension (A). Axial images from noncontrast MRA head, demonstrating an asymmetrically enlarged left occipital artery (arrowhead). Arterialized veins (arrows) are seen in the left occipital and temporal lobes (B, C). Lateral views of left occipital artery angiogram demonstrating a venous recipient pouch in the left transverse sinus wall (arrowhead) with retrograde cortical venous drainage (arrows) (D, E). Imaging consistent with high‐grade dural arteriovenous shunt. A 77‐year‐old woman presenting with swollen preseptal soft tissues, proptosis, conjunctival injection, and eye pain. Axial reconstructions of a CTA Head demonstrating right eye proptosis (yellow arrow) and an asymmetrically enlarged and enhancing superior ophthalmic vein (arrowhead). Lateral views of the right internal carotid angiogram show an indirect CCF, with opacification of the superior ophthalmic vein (arrowhead) and right Cavernous sinus (arrow), with the absence of the ipsilateral inferior petrosal sinus (IPS), including on the normal venous phase of the brain (star denotes expected location).

Dural arteriovenous shunts (DAVS) are classically acquired lesions, accounting for approximately 15% of all intracranial vascular malformations and generally present later in life.^33^ They are defined by their arterial supply from external carotid artery branches, often meningeal branches, and are drained by dural sinuses, meningeal (dural), or subarachnoid (cortical) veins. The most common location, outside of the cavernous sinus, is the transverse and sigmoid sinuses (Figure 2A‐E).^33^, ^34^, ^35^. Although the exact etiology underlying this malformation remains unclear, the role of dural sinus thrombosis leading venous hypertension (either local or general) with subsequent recanalization allowing for direct arterial shunting into the sinus, is one proposed mechanism.^36^, ^37^

Two major classification systems for DAVS are the Cognard and Borden systems, which are based on the cortical venous reflux.^33^, ^36^, ^38^ Cortical venous reflux is the determinant (in both classification systems) of the whether the lesion is high‐grade (presence of cortical venous reflux, associated with risk of ischemia, hemorrhage, death etc.) or low grade (minimal risk of hemorrhagic stroke, venous ischemia, or death). Clinical presentations of these lesions can also differ based on the location of the lesion (deep vs. superficial, supratentorial vs. infratentorial venous drainage) and size of the malformation. DAVS are seen more frequently in the 5th‐ 7th decades of life. A common presentation of DAVS at the skull base is pulsatile tinnitus due to the common location of transverse or sigmoid sinus.^33^, ^34^, ^36^ However, the most common location of a DAVS is within the cavernous sinus (Carotid cavernous fistula, 40‐60%),^39^ where it is associated with numerous ophthalmologic symptoms discussed in the next section. For lesions outside of the cavernous sinus, headache, seizures, intraparenchymal, and subarachnoid hemorrhage as well as edema/mass effect can be seen depending on the location, drainage, size, and pace of lesion progression.^33^, ^34^, ^40^

Diagnosis of these lesions is made by CT angiogram (CTA), MR angiogram (MRA), and conventional angiogram. A case study of 27 patients demonstrated a sensitivity of 91% using MRA versus the gold standard for conventional angiogram.^41^ A key to imaging evaluation is to characterize the arterial feeders and to map out the recipient vein and its venous outflow – critical to evaluate if retrograde venous drainage is present.^34^ Furthermore, the presence of retrograde cortical venous drainage and venous ectasia substantially increase the risk for bleeding (from 3% to 27% per annum increase).^34^, ^37^, ^41^ To eliminate the risk of bleeding, the recipient vein or venous pouch must be completely oblitrated.^33^ The primary therapeutic intervention is endovascular therapy.^33^, ^39^, ^42^ Similar to other AVS, there are multiple techniques available including access point and device choice.^43^ Placement of micro coils, liquid embolic agents and particulates can be used depending on size and location of lesion. These techniques demonstrate a high rate of complete fistula obliteration ranging from 70% to 90%. If determined to follow conservatively, surveillance imaging should be employed at intervals, and with any change in symptoms.^35^, ^42^, ^44^

#### Carotid cavernous fistulas

Carotid cavernous fistulas (CCFs) are a variant of DAVS with certain anatomical features. CCFs are defined by an abnormal communication between the cavernous sinus and carotid arterial system (Figure 2F‐J).^45^, ^46^

There are multiple hypotheses as to the origin of these lesions. They are accepted as being acquired, following trauma or a response to thrombosis of the cavernous sinus and an attempt to recanalize venous outflow.^45^ There is a female predominance (usually postmenopausal) in this condition, possibly suggesting a hormonal connection. Predisposition for these lesions overlaps with most vascular pathologies – direct trauma, hypertension, atherosclerotic disease, connective tissue disease, and possibly pregnancy.^45^

These lesions are classically divided in two subgroups of high and low flow lesions. In 1985, Barrow et al, developed a classification system for carotid cavernous sinus fistulas, Type A, B, C, and D. Type A lesions are high flow or direct, whereas B, C, and D are low flow or indirect lesions.^46^

In addition to high versus low flow, the route of drainage impacts presenting symptoms. Classic presentation of type A high flow direct lesions is a triad of exophthalmos, cephalic bruit and conjunctival congestion; however, symptoms depend on the characteristics of the lesions described below.^47^, ^48^ Additional symptoms included a variety of ophthalmologic findings, blurred vision, diplopia, facial pain, and headache. ^33^, ^49^, ^50^, ^51^ CCF’s can also involve cortical veins with retrograde drainage, compatible with a high‐grade DAVS, with high risk of intraparenchymal bleeding, ischemia, and focal neurological findings beyond the ocular symptoms.

The diagnostic approach should include a detailed ophthalmologic examination looking for evidence of increased intraocular pressure or direct observation of vascular pathology, and to provide a pretreatment ophthalmologic baseline. Neurovascular imaging with DSA is the gold standard for diagnosis. However, in a case series consisting of 53 patients, CTA was noted to be as good as DSA and performed better than MRA when lesions are located in the 4^th^ of 5^th^ segment of the ICA.^52^

Compared to other vascular malformations, the risk of spontaneous intracranial hemorrhage and mortality is exceptionally rare, unless cortical venous drainage is present. Consideration of treatment is in relation to ocular morbidity.^45^ An important consideration is that 20‐50% of low flow dural lesions will close spontaneously, therefore watchful waiting is a key component of treatment strategies.^51^ High flow, Type A, carotid cavernous fistulas generally lead to higher ocular morbidity, rarely close spontaneously, and therefore usually require intervention. Clinical indication for intervention includes progressive vision loss and eye movement abnormalities. If only mild symptoms are present, first‐line therapy involves targeting the elevated ocular pressure with topical medications. If conservative therapy is not successful or symptoms are severe endovascular therapy is the first‐line therapy with a range of 55‐99% success rate in the literature. If endovascular therapies fail or are not possible, the next steps may include surgery or stereotactic radiosurgery.

### Cerebral cavernous malformations

**Figure 3:**
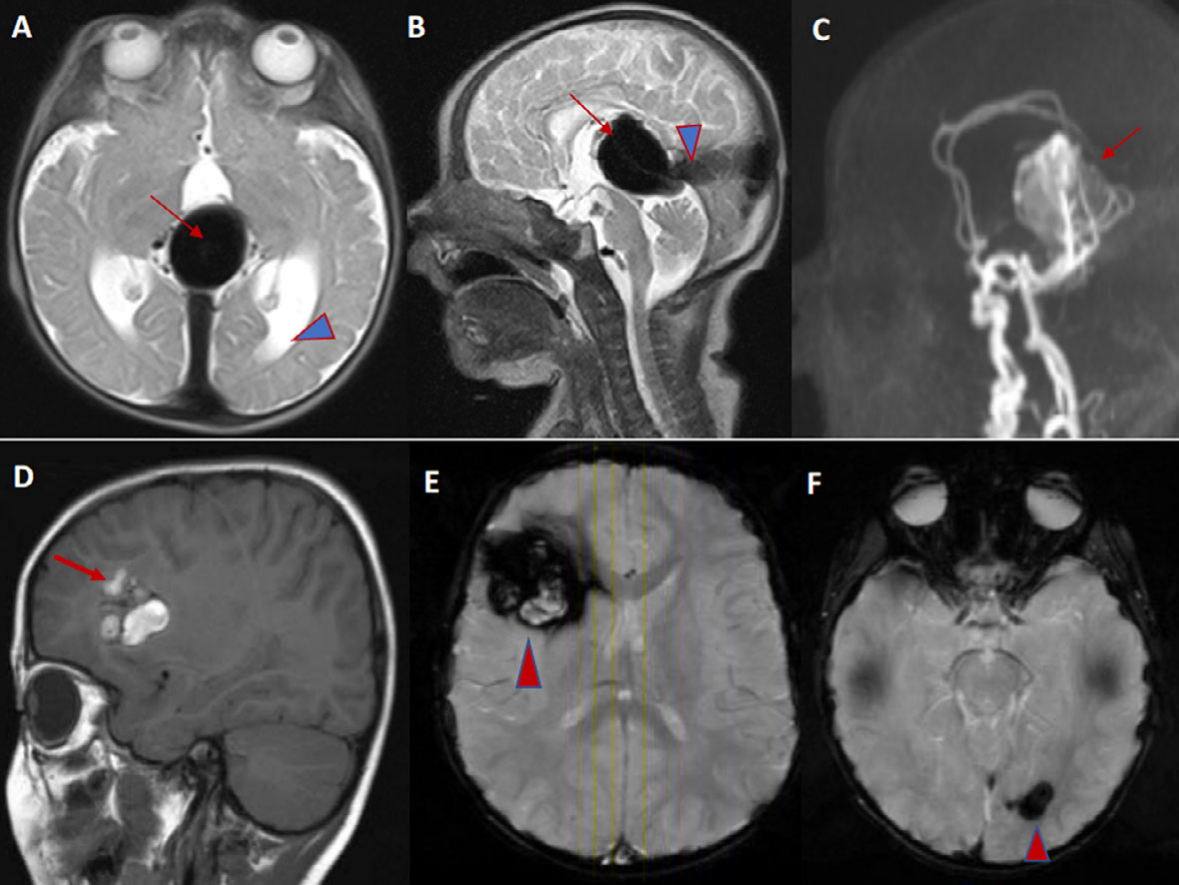
A 6‐day‐old boy with high output heart failure, pulmonary hypertension, and reversed diastolic aortic flow. Axial & Sagittal T2W MRI brain showing dilated medial prosencephalic vein (arrow) and persistent falcine sinus (arrowhead) (A, B). The visualized brain parenchyma is normal for age. Sagittal 3D reconstruction of noncontrast MRA head demonstrating arterial pedicles from the limbic arcade (arrows) supplying the vein of Galen malformation (C). Patient with familial Cavernoma. Axial GRE (blood‐sensitive) images demonstrating the classical “popcorn” appearance of a large right frontal lobe CM (arrow) with classical imaging findings of blood in different stages of breakdown (both high & low T2 signal, high and low T1 signal), hypointense rim on T2W images and surrounding vasogenic edema (D, E). Multiple foci of susceptibility in the right and left hemispheres of the brain (arrowheads) compatible with multiple cavernous malformations (F).

Cerebral Cavernous Malformations (CCMs) are also known as cavernous angiomas, cavernous hemangiomas, or cavernomas. These lesions are characterized as a cluster of sinusoidal vascular channels in which the vessel walls are thinner and less elastic than normal, and more prone to leaking and rupture.^53^, ^54^ In a study by Flemming et al including 2700 individuals aged 50‐90 year‐olds, incidence of CCM was 0.44%; other studies cite incidence of 0.1‐0.8%.^55^, ^56^ Lesions are categorized as sporadic or familial. Up to 20% of cavernous malformations are familial and are more likely to present with multiple lesions (Figure 3D‐F).^57^ The three associated genes CCM1, CCM2, and CCM3 are autosomal dominant and appear in all races with a higher incidence in Hispanic populations. The genetic underpinning of familial CCMs is important in angiogenesis and the permeability of the endothelium. The gross pathology and anatomic characteristics of sporadic and familiar are similar.^54^, ^56^, ^58^, ^59^

CCMs are frequently found in the supratentorial areas and seizure (up to ~50%) is the most common clinical presentation.^60^ Given the high incidence of seizure at presentation, the International League Against Epilepsy has created a grading system of likelihood of causality for these lesions including definite, probable, or cavernous unrelated to epilepsy.^61^ CCMs can also present with hemorrhage (~25%) and focal neurologic deficit without hemorrhagic changes. A large portion of CCMs is found incidentally (20‐50%).^53^, ^62^, ^63^

The annual risk of intracranial hemorrhage is predicted ~1‐2% – many studies have assessed variables to predict risk with mixed results. Female sex, size of lesion, multiple lesions, and location in the brain stem are associated with a higher risk of bleeding. Familial CCMs demonstrate a slightly higher risk of hemorrhage even when controlling for multiple lesions.^56^, ^64^ Two large observational studies with a total of 500 patients showed no increased risk of hemorrhage during pregnancy, delivery, or postpartum;^65^, ^66^, ^67^ however, patients with familial CCM should discuss preimplantation genetic testing and obtain MRI imaging prior to conception. In a meta‐analysis including more than 1300 patients with CCMs, taking antithrombotic was associated with lower bleeding risk. It is hypothesized that bleeding associated with CCMs is likely related to thrombus formation within the malformation.^68^

MRI imaging with gradient susceptibility imaging is recommended for evaluating CCMs.^57^, ^69^ The classic “popcorn” appearance is a typical image finding. The differential diagnosis for such lesions include cerebral amyloid angiopathy, hemorrhagic neoplasms, pleomorphic xanthoastrocytomas, and oligodendrogliomas.^55^, ^57^ In the right clinical context, MRI or CT angiography are sufficient for making the diagnosis. Conventional angiogram is not routinely indicated for diagnosis or characterization of the lesions, as these are classically angiographically occult.

Therapeutic strategies for cavernous malformations are largely based on symptoms and location. There are no large randomized clinical trials for resection versus observation for asymptomatic lesions. However, there are large observational studies to suggest consideration of surgical evaluation in patients with lesions in the brain stem, eloquent cortex, history of hemorrhage, and medically refractory epilepsy.^60^, ^70^, ^71^ Stereotactic radiosurgery is not routinely recommended as a treatment option. In addition, routine surveillance imaging without new symptoms is not routinely recommended as asymptomatic lesions are not routinely intervened on.^57^

### Telangiectasia

Brain capillary telangiectasia (bCT) tend to be clinically silent and their discovery is incidental. bCT are small dilated capillaries intermixed with normal brain parenchyma.^72^, ^73^ These lesions are most commonly found in or near the brain stem – most commonly in the pons.^73^ Depending on their location and size, bCT can present with cranial nerve palsies, weakness, seizures, or visual changes. The underlying etiology of these lesions is thought to be vascular endothelial dysfunction. There are reports of lesion formation postradiation.^74^ While bCT in isolation are relatively benign they can be seen in syndromes including Hereditary Hemorrhagic Telangiectasia (HHT).

Due to the slow flow and small nature of most lesions, they are often not appreciated on conventional imaging modalities including CT, conventional T1‐ and T2‐weighted MRI and also can be missed on digital subtraction angiography. T1 MR imaging with gadolinium enhancement and gradient echo images are valuable in this imaging modality of choice. The visibility on gradient echo imaging is likely related to the sluggish blood flow within this vascular malformation.^72^, ^73^

Treatment of brain capillary telangiectasias is largely conservative. There are limited case reports of resections of pontine lesions that are symptomatic. The use of anticoagulation or antiplatelet medication should not be withheld in patients with isolated telangiectasias, considerations of anticoagulation choice should be considered in patients with the diagnosis of HHT as described below.^75^

### Anatomic variants

#### Developmental venous anomalies

Developmental venous anomalies (DVAs) (referred to at times as venous angiomas, cerebral venous malformations, and cerebral venous medullary malformations) are low flow vascular malformations characterized by their “caput medusae” which are radially arranged configurations of medullary veins separated by normal white matter.^40^ In 2017, Mooney et all, reported that DVAs are the most common vascular malformation found on autopsy, with an incidence of 2.6% in a series of 4000 patients.^40^ There are multiple proposed hypothesis for the etiology of these lesions. One leading hypothesis postulates that they are formed during intrauterine life as a response to ischemia and venous hypertension. Critically, DVAs drain normal brain tissue, functioning like normal veins.

Most DVAs are benign lesions. Symptoms are typically related to thrombosis, with an incidence of hemorrhage or infarct between 0.22 and 0.68% per year.^76^, ^77^ The mechanism for both is likely related to acute thrombus in the collecting vein. These lesions are discovered incidentally most of the time, if symptomatic, most common symptom is headache followed by seizures, focal motor, or sensory impairments.^40^, ^78^, ^79^, ^80^ The association between CCMs and DVAs is clinically important because while most DVAs are asymptomatic benign lesions, but when associated with CCMs the incidence of hemorrhage from the CCM can significantly increase.^81^, ^82^ DVAs are commonly found in association with CCMs in particular when DVAs are infratentorial and they are multiple. This data might suggest a potential for the transformation of DVAs to CCMs over time.^83^

**Figure 4:**
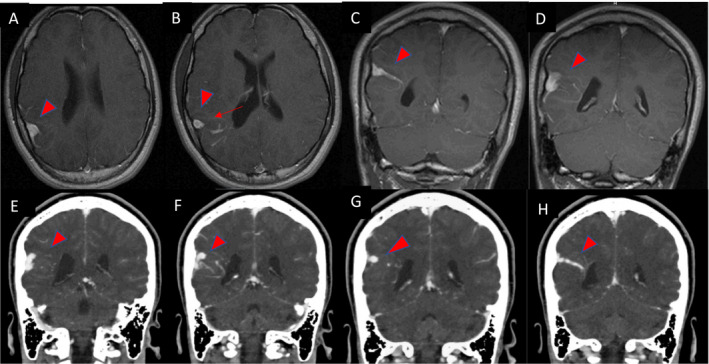
Axial postcontrast T1W MRI demonstrating a typical dural venous anomaly (arrowheads) (A,B,C,D) with a “medusa’s head (arrow) (B). Coronal images from a CT with venous phase, demonstrating the dural venous anomaly (arrowheads) extending from the ependymal surface to the cortical surface (F,G,H,I).

DVAs may be seen on most imaging modalities (Figure 4). On noncontrast CT, associated hemorrhage, and calcification can be seen. CT venogram can reveal the characteristic “caput medusae” and the draining vein. MRI with gradient‐echo T2‐weighted images is used to evaluate associated vascular abnormalities such as CCMs in addition to assess for any parenchymal abnormalities.^80^, ^84^, ^85^ Given the low flow nature of these lesions, DSA is not routinely utilized, however, it is still the gold standard to assess for flow dynamics prior to intervention.

The main therapeutic approach to DVAs is observation, given their function as normal venous channels, unless presenting with bleeding, infarct, or mass effects. Thrombosed DVAs should be treated as a cerebral venous thrombosis and may require systemic anticoagulation. Surgical or endovascular therapies must be pursued with great caution as venous infarction can occur after resection of the lesions. Hence, intervention is generally reserved for intractable seizures or symptoms.^86^, ^87^, ^88^

#### Vein of galen malformations

Vein of Galen Malformation also known as Aneurysm of the Vein of Galen is a congenital lesion that develops when multiple arteriovenous shunts form between the choroidal/limbic circulation and a persistent embryologic vein (median prosencephalic vein of Markowski), leading to venous enlargement and (incorrect) characterization as a Vein of Galen Aneurysm.^89^, ^90^, ^91^ The classification of these malformations is based on the arterial connections, either direct arterial connections or many smaller choroidal feeders.^92^, ^93^

Clinical presentations are usually divided into three (age‐related) categories: (1) Neonatal presentation – 2^nd^ most common, (2) Infantile/childhood presentation – most common presentation, and (3) Late childhood or adult presentation. A general rule is the earlier the presentation the more severe the symptoms.

Neonatal presentation – Presentation at this age is due to high AV shunting, causing high‐output cardiac failure, very commonly associated with pulmonary hypertension, and in the most severe cases with poor descending aortic flow and visceral organ ischemia (Figure 3A‐C).

Infantile/early childhood presentation – Macrocephaly, hydrocephalus (due to hydrodynamic inadequate resorption of CSF), delayed neurological milestones, prominent scalp/facial collateral veins, and possibly papilledema are the most common signs and symptoms.

Older childhood/early adulthood presentation – Least common. The presentation and natural history are that of deep AVM’s with deep venous drainage. These will present with intraparenchymal or intraventricular hemorrhage, headache, and seizure.^94^

Lasjaunias et al., had a landmark paper in 2006 outlining a proposed a 21‐point scoring system, the “Bicetre neonatal evaluation score for VGAM”, to determine urgency of treatment and describing the group’s experience.^90^ Endovascular therapy is the treatment of choice for these lesions. In a case series of 233 patients in 2006, the authors highlighted that the treatment of these lesions is associated with a 10% mortality, however, 74% of surviving patent was reported to be neurologically normal, emphasizing that complete obliteration of the lesion is not always necessary in the neonatal and infantile types.^90^, ^95^ Children or adults presenting with a vein of galen malformation can be medically managed depending on symptom and systemic consequences.^96^

#### Sinus peri‐cranii

Sinus peri‐cranii is an abnormal connection between intracranial and extracranial vasculature presenting during childhood as a nontender palpable scalp lesion, usually frontal midline location. Most patients remain asymptomatic although cosmetic issues can be a major reason for seeking medical attention. Other symptoms include headaches and tinnitus. Sinus peri‐cranii can be congenital or secondary to trauma. Congenital cases, similar to DVAs, are hypothesized to be related to venous hypertension during the intrauterine period. DSA is required to assess the flow dynamics which determines timing and type of treatment if needed.^97^, ^98^

## Spinal cord vascular lesions

### Arteriovenous shunts

**Figure 5:**
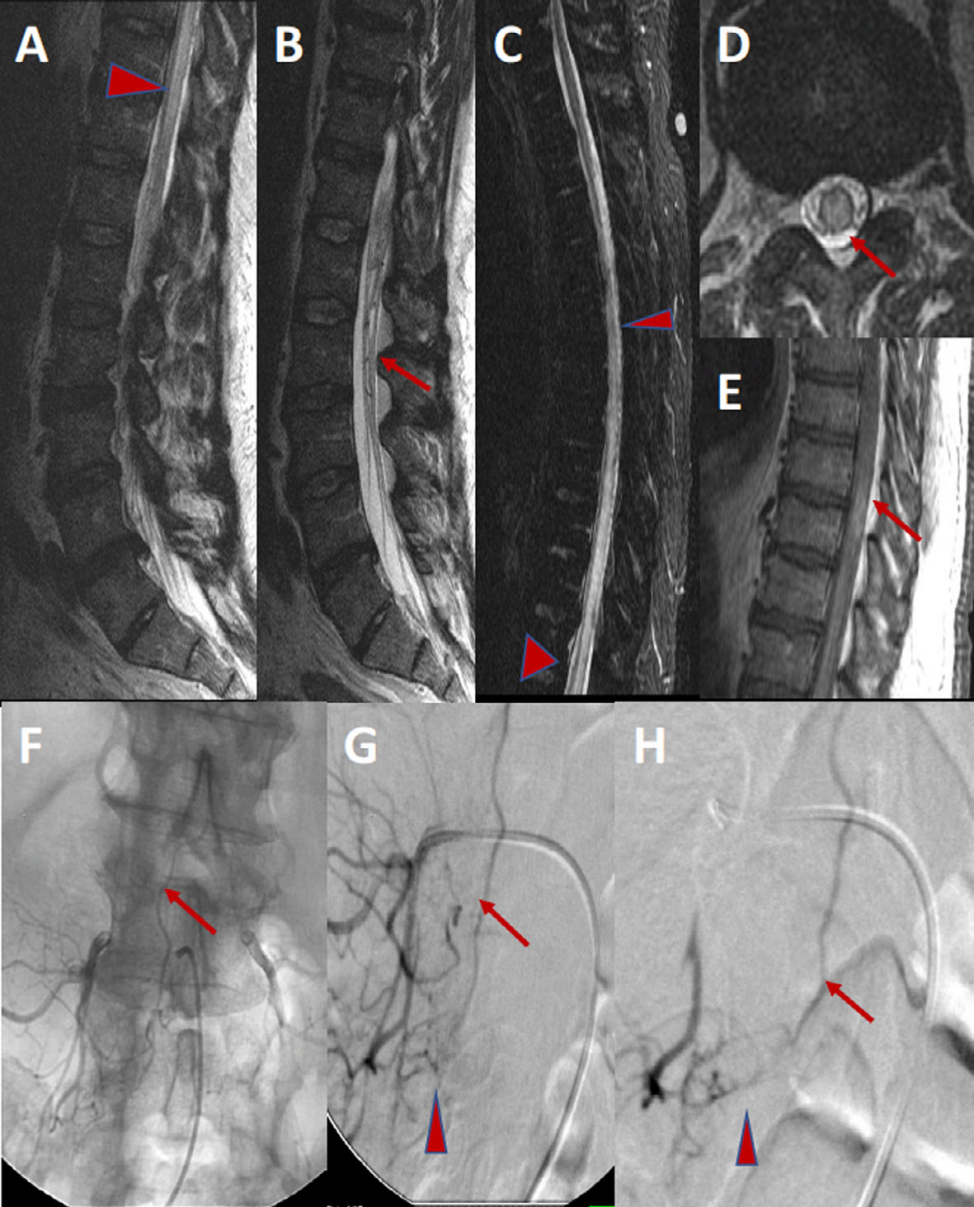
A 60‐year‐old man presenting with a 1‐year history of increasing lower extremity weakness, and now with occasional bladder and bowel incontinence. Sagittal MRI T2W images of the left spine demonstrating abnormal increased T2 signal in the cord (arrowhead) and serpiginous vascular structure (arrow) interspersed among the normal nerve roots (A, B). Sagittal MRI STIR image of thoracic spine demonstrating the significant extent of the cord edema (arrowheads) (C). Axial MRI T2W image, demonstrating abnormal increased T2 signal, primarily affecting the central grey matter (arrow) (D). Sagittal postcontrast T1W image demonstrating patchy enhancement of the spinal cord (arrow) (E). AP un‐subtracted and subtracted angiography of the right L4 segmental artery demonstrating the point of AV shunting (arrowhead) in the neural foramen, and the retrograde venous drainage in the enlarged radiculomedullary vein extending cranially to the spinal cord (arrow) confirming the diagnosis of dural arteriovenous shunt (F, G). Subtracted image postembolization with NBCA (glue) demonstrating the glue cast filling the point of AV shunting (arrowhead) and the draining vein (arrow), necessary for achieving cure (H).

**Figure 6:**
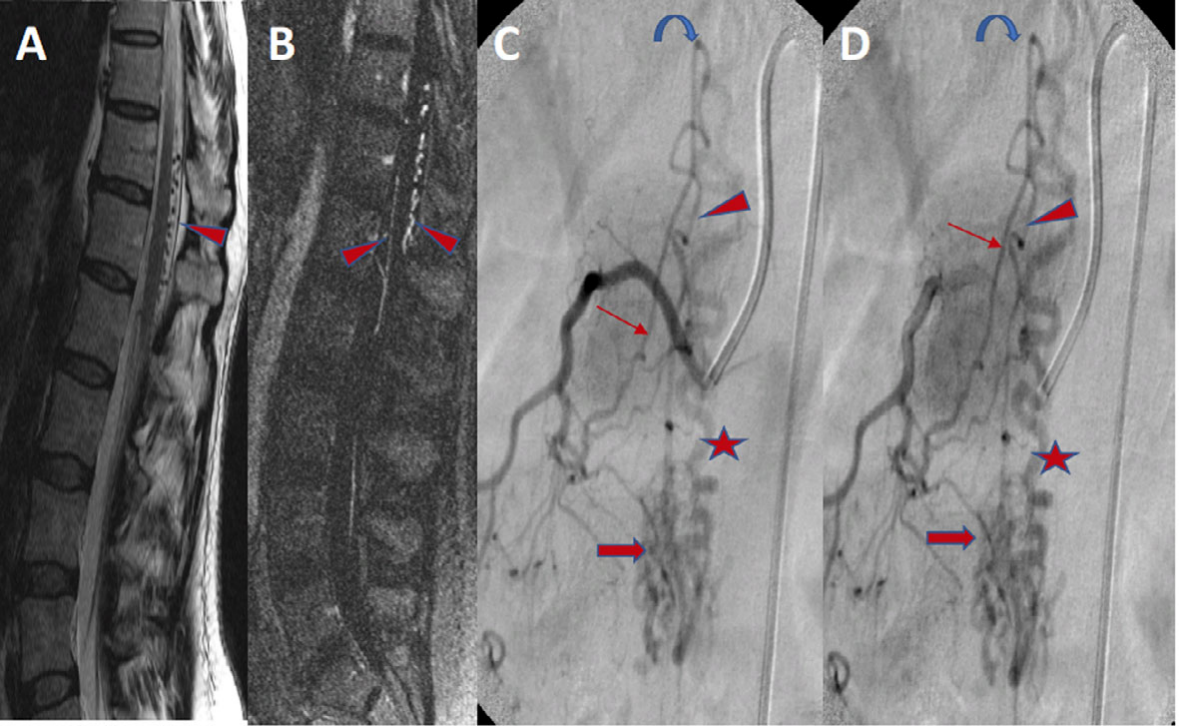
A 40‐year‐old man presenting with lower extremity radicular pain. Sagittal T2W MR left spine showing normal cord signal, but abnormal vascular flow voids along the dorsal surface of the lower spinal cord (arrowhead) (A). High‐resolution postcontrast T1W view of the left spine demonstrating abnormally enlarged and serpiginous enhancing vessels along the ventral and dorsal surfaces of the cord (arrowheads) (B). AP views subtracted from a DSA injection of the right T12 intercostal artery, demonstrating an enlarged radiculopial artery (arrow) extending to the anterior spinal artery (ASA) (arrowhead), with a classic hairpin turn (curved arrow). The ASA is slightly enlarged and extends to the point of AV shunting at the level of the conus medullaris (fat arrow). The abnormally dilated and enlarged draining vein is seen extending along the dorsal surface of the cord (star) (C, D). Image findings consistent with pial arteriovenous.

Arteriovenous shunts are the most common type of spinal cord vascular malformations. Still these malformations are quite rare and more frequently detected during childhood and young adulthood. Spinal cord vascular malformations have been classified into the following categories; dural AV fistula (type I), glomus arteriovenous malformations (type II), juvenile metameric arteriovenous malformations (type III), spinal pial AV fistula (type IV). Table 2 summarizes the main features of each malformation. Among the spinal AV shunts, neurologists most frequently encounter cases of dural AV fistula (type I) which accounts for the majority of spinal cord AVS. These malformations are acquired, and characterized as low‐pressure shunts commonly located in the dural sleeve of dorsal nerve root of the lower thoracic spinal cord (Figure 5).^99^ Timely recognition and management of this pathology has significant prognostic implications although the diagnosis can be challenging. Figure 6 demonstrates a case of pial AV fistula. A typical clinical scenario is a middle aged or older male presenting with slow progressive asymmetric sensorimotor symptoms treated for neuropathy for years with recent worsening of symptoms and development of bowel, bladder, and sexual dysfunction. These patients are frequently treated for inflammatory myelopathy processes with no response or worsening of the symptoms after receiving intravenous steroids.^100^ Standard spinal cord MRI with contrast shows intramedullary T1 hypointensity, T2 hyperintensity extending over multiple vertebral levels with flow voids.^101^ Similar to many other vascular malformations, DSA is the gold standard diagnostic modality, although spinal cord MRA can localize these lesions in about 80% of cases.^102^ Endovascular intervention with liquid embolic agents and open surgical interventions are both well‐established for the management of these malformations. Definitive treatment requires occlusion of the recipient (radiculomedullary) vein. Endovascular therapy is associated with shorter hospital stay and complications while surgical interventions lead to more durable clinical outcomes in fistulas with multiple feeding vessels.^103^ Significant clinical improvement is the rule with treatments with either method, particularly if symptoms have been limited in duration. The main predictor for postintervention recovery is the preintervention clinical status. Nonetheless, favorable outcomes are commonly seen even in patients with long‐standing and severe symptoms.^104^

**Table 2:** Classification and features of spinal arteriovenous shunts.

Type	Characteristics and location	Presentation	Treatment	Outcomes
Dural AV fistula (type I)	Direct fistula in dural sleeve of nerve root	Myelopathy (venous hypertension)	Surgery > endovascular embolization	Reversible symptoms with intervention
Glomus AV malformations (type II)	Intramedullary, pial AVM	Myelopathy and hemorrhage	Surgery +/‐ endovascular. Radiosurgery in some cases.	Reduction in symptoms after intervention
Juvenile metameric arteriovenous malformations (type III)	Intra and extra medullary paraspinal juvenile AVM	Myelopathy and hemorrhage	Surgery + endovascular (palliative)	Intervention halts the progression of neurological symptoms and minimizing neurological sequelae
Pial AV fistula (type IV)	Intradural, extra medullary direct fistula	Myelopathy in low flow, hemorrhage in high flow	Surgery (low flow), endovascular +/‐ surgery in high flow	Resolution of symptoms with intervention

### Hypervascular neoplastic lesions: Hemangioblastomas

**Figure 7:**
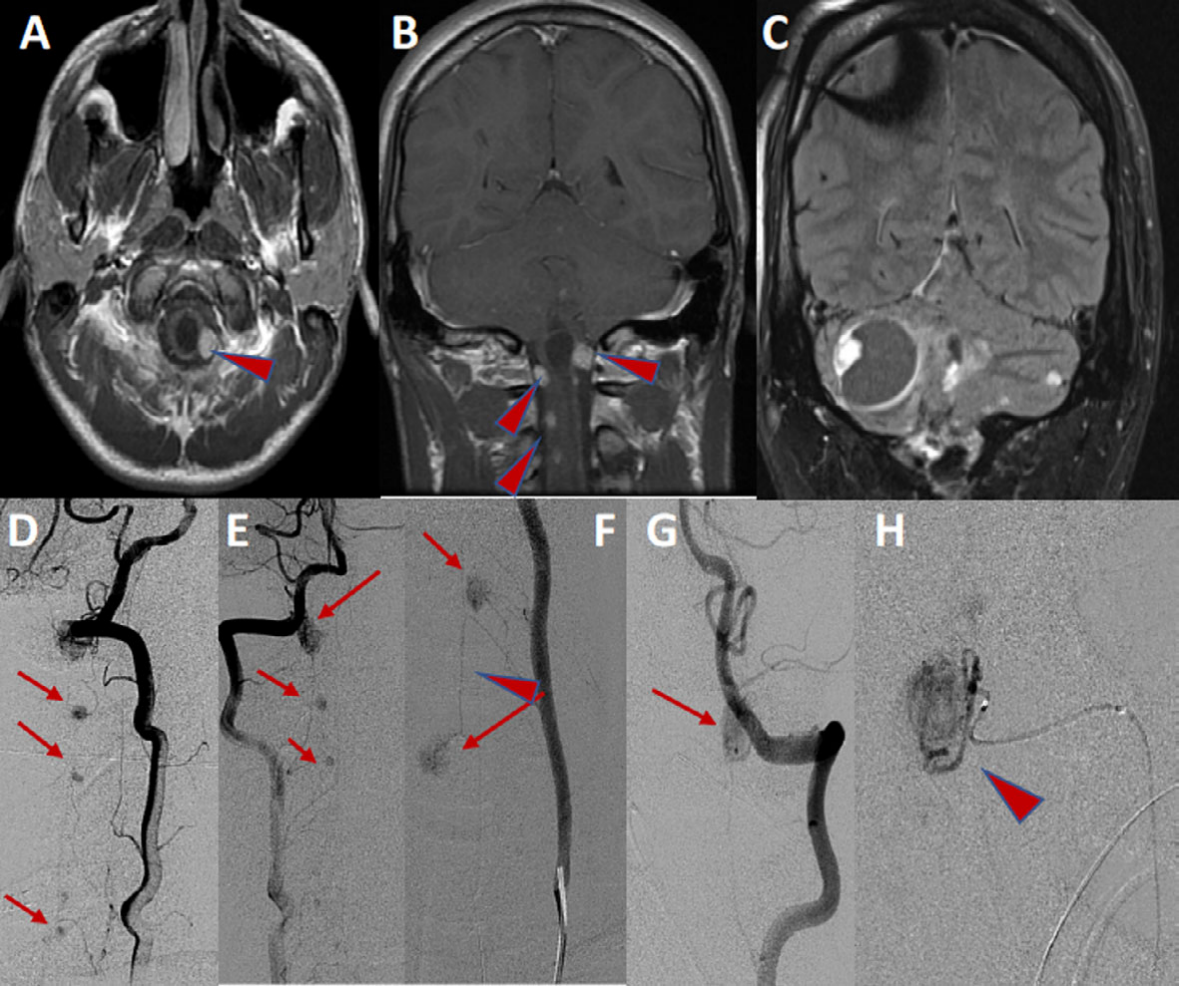
MRI axial and coronal postcontrast T1‐weighted images demonstrating multiple enhancing intra‐axial cerebellar nodules and nodules on the pial surface of the spinal cord (arrowheads) (A, B). MRI coronal postcontrast T1W image demonstrating classic “cyst & mural nodule appearance” of right cerebellar lesion (C). Vertebral artery DSA demonstrating multiple hypervascular nodules (arrows) along the cervical cord consistent with hemangioblastomas (D, E). Left vertebral artery injection with multiple hemangioblastomas, with ASA (arrowhead) supply (F, G). Figure H shows microcatheter injections of segmental artery supply to larger hemangioblastoma at the Craniocervical junction.

Hemangioblastomas are slow‐growing and highly vascularized tumors found mainly in the posterior fossa but also as a spinal cord lesion. Hemangioblastomas are usually intramedullary (mainly located in the dorsal column) and predominantly present with sensory symptoms and back pain (Figure 7). Hemangioblastomas occur as sporadic lesions in the majority of cases, although up to 20‐30% of cases can be secondary to von Hippel–Lindau (VHL) syndrome.^105^ Hemorrhagic complications, such as subarachnoid hemorrhage, are rare. On spinal angiography, they appear with an enhancing nidus associated dilated arteries and prominent draining veins. Surgical resection with or without prior endovascular embolization is the only therapeutic option.^106^

### Cavernous malformations

Spinal cord cavernous malformations are rare intramedullary lesions with various forms of presentations. While a group of these patients report mild, chronic and stable neurological symptoms such as pain, weakness, and paresthesia, others can present with acute or intermittent episodes of neurological symptoms. There is a slight female predominance and the peak age for identification of these lesions is around the fourth decade of life. The thoracic spine is the most common location. Acute debilitating symptoms are usually associated with hemorrhages and once a hemorrhage occurs the risk of re‐bleeding can be as high as 66% per year.^107^ Such a high risk of recurrent hemorrhage warrants early surgical intervention and removal. On MRI, cord edema is usually not appreciated until a hemorrhagic event occurs where a mild degree of postcontrast enhancement is frequently seen.

## Syndromes

**Figure 8:**
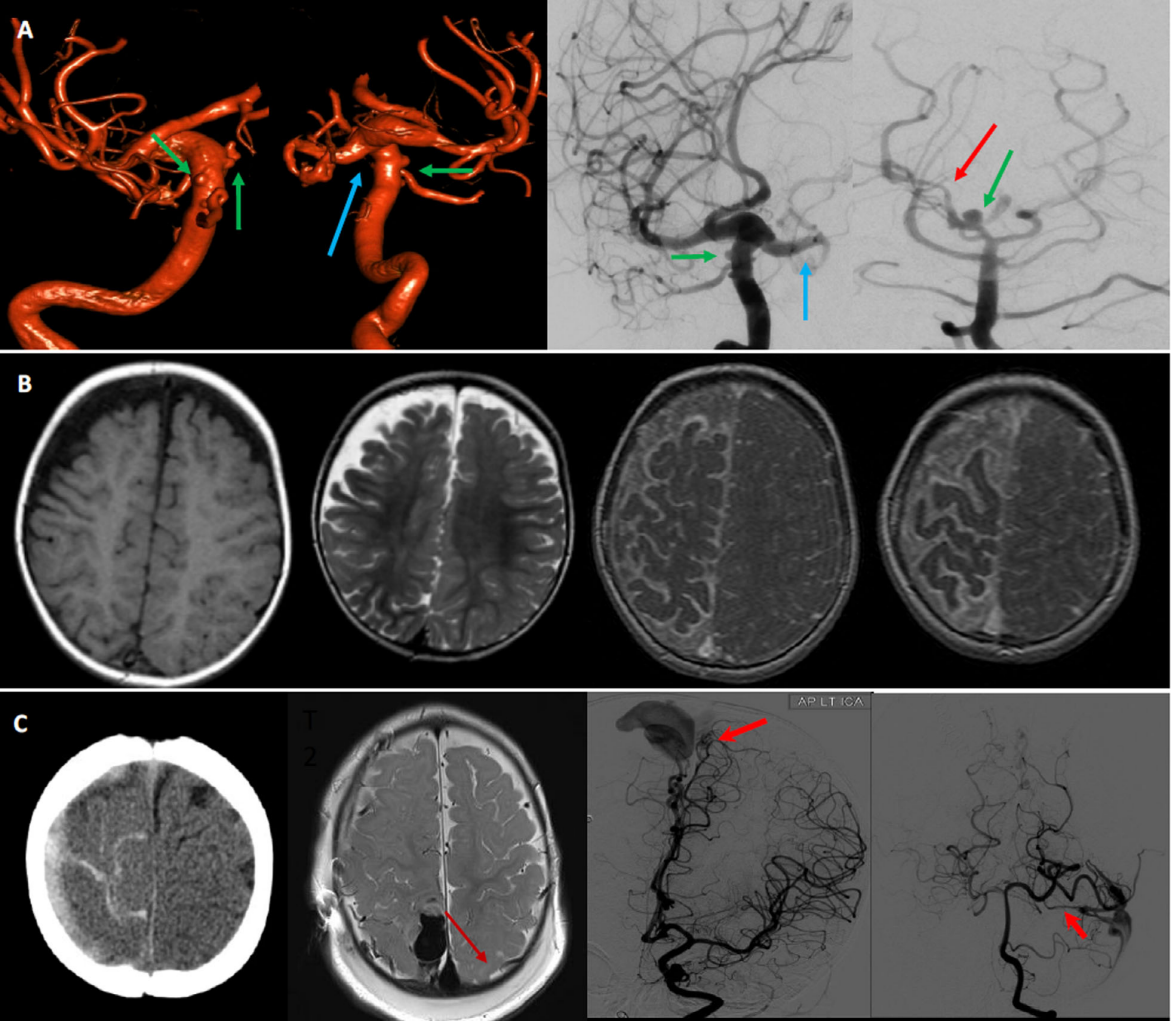
A: 14‐year‐old girl with evidence for arterial abnormalities on standard screening studies. 3D reconstruction of angiography from a right internal carotid artery injection demonstrating multiple small aneurysms (green arrows) arising from a dysplastic appearing supraclinoid region. In addition, focal dysplastic widening of the right posterior communicating artery (blue arrow), small right posterior cerebral artery aneurysm (green arrow), and dysplastic narrowing of the right posterior cerebral artery were observed. B: A 4‐year‐old patient with Sturge‐Weber syndrome. Brain MRI demonstrates asymmetric volume loss (T1), calcification (T2 signal), and abnormal enhancement (pial angiomatosis) in the sulci and surface of the right cerebral hemisphere (T1 postcontrast). C: A previously healthy toddler with acute onset apnea and choking. Noncontrast CT scan demonstrating subarachnoid hemorrhage and subdural hematoma. Axial T2W MRI brain, with abnormal vascular structure (recipient venous pouch) along medial margin of right parietal lobe (arrow). Aangiography demonstrating the enlarged right anterior cerebral artery supplying the right parietal arteriovenous shunt with dilated recipient venous pouch (arrows). AP left vertebral angiogram demonstrating a separate posterior fossa arteriovenous shunt (arrow).

CNS vascular malformations are frequently diagnosed in the setting of systemic diseases and syndromes with multiorgan presentations. There are several known familial or sporadic syndromes that exhibit as vascular tumors or malformations. Unlike isolated vascular lesions, these syndromes are difficult to treat and can have a progressive detrimental course. Early identification of these syndromes allows for closer surveillance and early management and possible prevention of catastrophic cerebrovascular events.^108^ A variety of rare syndromic vascular malformations exists. In this section, we provide an overview of major syndromes with CNS vascular presentations (Figure 8).

### Hereditary hemorrhagic telangiectasia (HHT)

HHT (also known as Osler–Weber–Rendu disease) is an autosomal dominant genetic condition associated with different genes including ACVRL1, ENG, and SMAD4 presenting with multiple AVS. The most common presentation is recurrent epistaxis and subsequent evaluations reveal skin and mucosal telangiectasias. Lung, liver, and brain are the key organs that can be affected.^108^ Cerebral AVMs occur in approximately 10% of individuals with HHT. Spinal cord AVMs are less common. The most common presentation is intracranial hemorrhage. The annual risk of an AVM rupture in HHT patients has been estimated to be 0.36% to 1.02% per year.^109^ Brain AVMs can evolve through the first and second decades of life and children with HHT need to be screened after puberty. Other vascular malformations, such as cavernoma or DAVS can be present in these patients as well. In contrast to nonsyndromic AVMs, HHT related CNS AVMs are multifocal in as many as 50% of cases (Figure 5).^23^ While no clinical trial data are available to compare observational approach versus surgical intervention, data from the Brain Vascular Malformation Consortium HHT project showed a relatively low risk and favorable long‐term outcome (without reaching statistical significance) for surgical resection of brain AVMs in HHT patients. Generally, it is recommended that patients without symptoms or mild symptoms like headaches be monitored clinically with neuroimaging and surgical interventions will remain a viable option for those with severe symptoms such as seizure or hemiparesis.^110^

### Capillary malformation–arteriovenous malformation (CM‐AVM) syndrome

**Figure 9:**
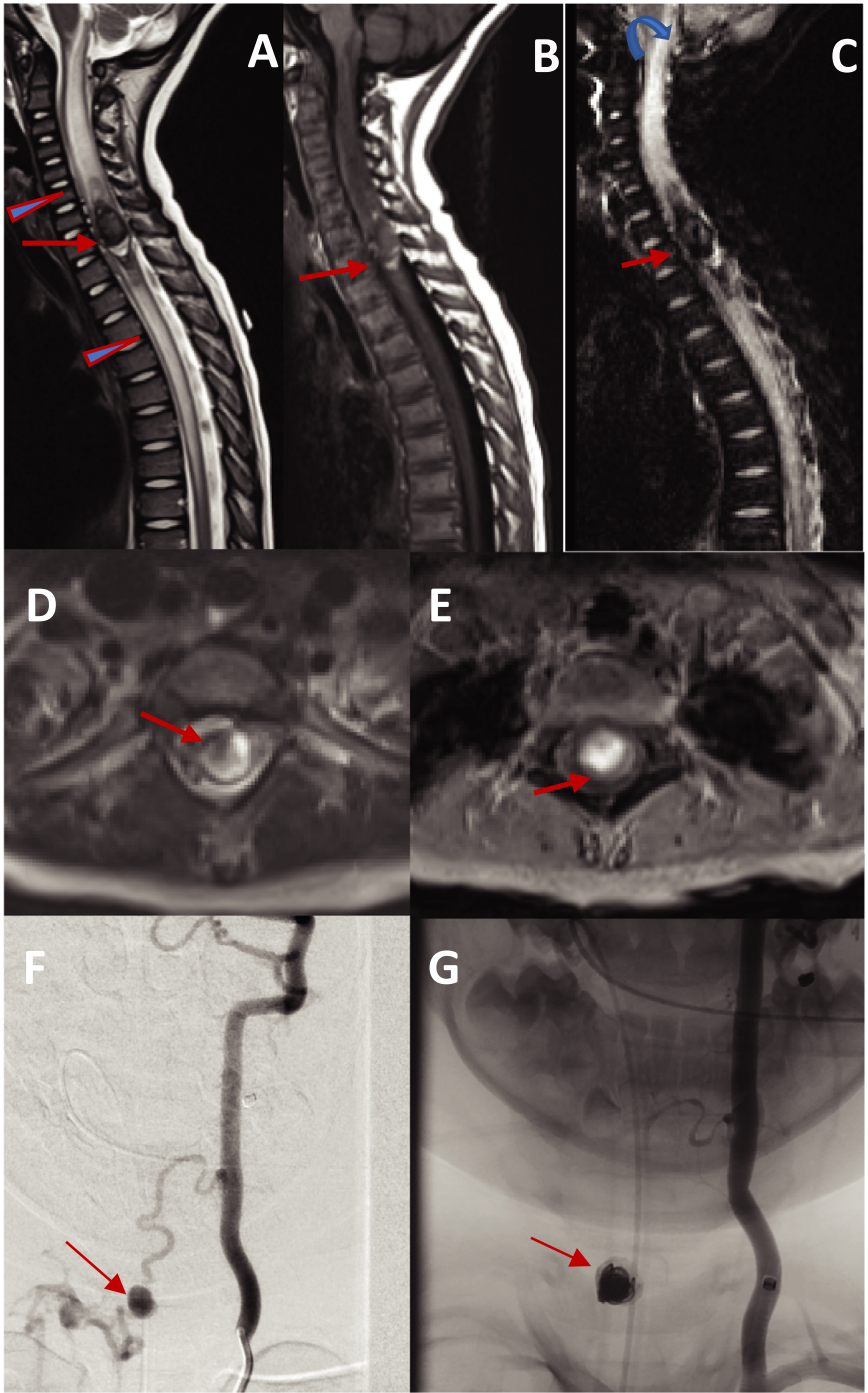
A 4‐year‐old girl presenting with acute onset quadriparesis and sensory deficit. Found to have Type IV cervical pial AVF with CM‐AVM syndrome (RASA 1 mutation): acute onset quadriparesis and sensory deficit. Sagittal T2W, T1W, and GRE MRI images of the cervical cord demonstrating long segment edema (arrow heads) in the cord, as well as a hemorrhagic lesion (arrows) at the C7/T1 level (A, B, C). Subarachnoid hemorrhage is seen as well (curved arrow) (C). Axial T2W &T1W images at C7/T1 showing products of hemorrhage (low T2, high T1, arrows) (D,E). Left vertebral artery injection demonstrating a Type IV C pial AVF with a recipient intramedullary venous varix as the site of hemorrhage (arrow). Roadmap image demonstrating microcatheter placement for embolization of the AVF (F, G).

CM‐AVM syndrome is characterized by the presence of multifocal small capillary malformations mostly seen on the face and limbs. A group of patients with this syndrome have associated AVMs and/or AV fistula in their brain. CM‐AVM syndrome is an autosomal dominant condition associated with EPHB4 or RASA1 genes. As a screening measure, brain imaging is recommended in all patients with CM‐AVM syndrome (Figure 9).^111^ Symptomatic AVMs and AVF are treated similar to nonsyndromic lesions.

### PHACES

Posterior fossa malformations, hemangiomas, arterial anomalies, cardiac defects, eye abnormalities, and sternal or ventral defects (PHACES) syndrome are distinct clinical entities with cutaneous hemangioma as a hallmark feature. CNS vascular anomalies happen in more than one‐third of these patients and can be in different forms such as cerebral vessels agenesis/hypoplasia, persistent embryonic vessels, vessel occlusion and stenosis, dolichoectasia, and hemangioma. In addition, between 43% and 90% of patients with PHACES have a CNS structural abnormality leading to developmental delay, seizure, amorphism, and hydrocephalus.^112^ The vascular abnormalities (most commonly tortuosity) are usually ipsilateral to the facial hemangioma and often involve the carotid or vertebro‐basilar system in a segmental fashion. Vascular changes can progress to severe vasculopathy (mainly in the form of arteriopathy) and lead to cerebrovascular re‐organization similar to Moya‐moya disease (Figure 4B).^113^

### Vascular metameric syndrome

This syndrome encompasses a number of different vascular pathologies which, share an embryologic concept that an anomaly in one body segment simultaneously causes failure of nearby nerves, skin, and blood vessels. This syndrome can present with different clinical findings including cerebral arteriovenous metameric syndrome (CAMS) and spinal arteriovenous metameric syndrome (SAMS) or Cobb syndrome. The major imaging finding of vascular metameric syndrome is an arteriovenous malformation or fistula in the cerebrospinal area or the head and neck region.^114^ Cutaneous lesions can be hints to the location of vascular malformations in this syndrome. For instance, in SAMS or Cobb syndrome cutaneous vascular malformations are seen at a level lower than that of an arteriovenous malformation or fistula involving the spinal cord.^108^

### e. Sturge weber syndrome

Sturge–Weber syndrome (SWS), or encephalotrigeminal angiomatosis, is a nonhereditary condition with typical unilateral facial angioma (port‐wine stain) localized in the first and less often in the second and third sensory distribution of the trigeminal nerve. This condition is associated with leptomeningeal venous malformation of the ipsilateral parieto‐occipital and less frequently frontal regions as well as eyes (Figure 4C).^115^ Due to vascular malformations, the cortical and white matter perfusion is altered in SWS leading to brain structural changes and contributing to a higher risk of epilepsy, hemiparesis and stroke‐like symptoms, visual impairments, and severe migraines.^116^ Eye involvement with the vascular malformation can result in glaucoma and subsequent vision loss. Low‐dose Aspirin therapy has been implicated in reducing the risk of paroxysmal events such as migraine and stroke‐like events although controlled trials are lacking.^117^

## Summary and future directions^7^

CNS vascular malformations are a heterogeneous group of neurovascular conditions with diverse presentations, clinical course, and complications. With advancement in neurovascular imaging and genetic studies, an increasing number of individuals are being diagnosed in the presymptomatic phase. Understanding the underlying pathophysiology and differentiating vascular malformations from mimics are the key factors for success in early recognition, timely treatment, and systematic surveillance in such patients. Future studies are needed to advance our understanding on patho‐biology of CNS vascular malformations in order to develop robust prognostic models for the stratification of high‐risk individuals towards applying personalized interventions.
